# Adenosine, lidocaine and Mg^2+^ improves cardiac and pulmonary function, induces reversible hypotension and exerts anti-inflammatory effects in an endotoxemic porcine model

**DOI:** 10.1186/s13054-014-0682-y

**Published:** 2014-12-11

**Authors:** Asger Granfeldt, Hayley L Letson, Geoffrey P Dobson, Wei Shi, Jakob Vinten-Johansen, Else Tønnesen

**Affiliations:** Department of Anesthesiology, Aarhus University Hospital, Nørrebrogade 44 building 21 1st floor 8000, Aarhus, Denmark; Department of Anesthesiology, Regional Hospital of Randers, Skovlyvej 1, 8930 Randers, Denmark; Heart, Trauma & Sepsis Research Laboratory, Australian Institute of Tropical Health and Medicine, School of Medicine and Dentistry, James Cook University, Pharmacy and Medical Research Building 47, Rm 113B Townsville, Queensland Australia; The Cardiothoracic Research Laboratory, Carlyle Fraser Heart Center, Emory University School of Medicine, 387 Technology Circle Suite 180, Atlanta, Georgia 30313 USA

## Abstract

**Introduction:**

The combination of Adenosine (A), lidocaine (L) and Mg^2+^ (M) (ALM) has demonstrated cardioprotective and resuscitative properties in models of cardiac arrest and hemorrhagic shock. This study evaluates whether ALM also demonstrates organ protective properties in an endotoxemic porcine model.

**Methods:**

Pigs (37 to 42 kg) were randomized into: 1) Control (n = 8) or 2) ALM (n = 8) followed by lipopolysaccharide infusion (1 μg∙kg^-1^∙h^-1^) for five hours. ALM treatment consisted of 1) a high dose bolus (A (0.82 mg/kg), L (1.76 mg/kg), M (0.92 mg/kg)), 2) one hour continuous infusion (A (300 μg∙kg^-1^ ∙min^-1^), L (600 μg∙kg^-1^ ∙min^-1^), M (336 μg∙kg^-1^ ∙min^-1^)) and three hours at a lower dose (A (240∙kg^-1^∙min^-1^), L (480 μg∙kg^-1^∙min^-1^), M (268 μg∙kg^-1^ ∙min^-1^)); controls received normal saline. Hemodynamic, cardiac, pulmonary, metabolic and renal functions were evaluated.

**Results:**

ALM lowered mean arterial pressure (Mean value during infusion period: ALM: 47 (95% confidence interval (CI): 44 to 50) mmHg versus control: 79 (95% CI: 75 to 85) mmHg, *P* <0.0001). After cessation of ALM, mean arterial pressure immediately increased (end of study: ALM: 88 (95% CI: 81 to 96) mmHg versus control: 86 (95% CI: 79 to 94) mmHg, *P* = 0.72). Whole body oxygen consumption was significantly reduced during ALM infusion (ALM: 205 (95% CI: 192 to 217) ml oxygen/min versus control: 231 (95% CI: 219 to 243) ml oxygen/min, *P* = 0.016). ALM treatment reduced pulmonary injury evaluated by PaO_2_/FiO_2_ ratio (ALM: 388 (95% CI: 349 to 427) versus control: 260 (95% CI: 221 to 299), *P* = 0.0005). ALM infusion led to an increase in heart rate while preserving preload recruitable stroke work. Creatinine clearance was significantly lower during ALM infusion but reversed after cessation of infusion. ALM reduced tumor necrosis factor-α peak levels (ALM 7121 (95% CI: 5069 to 10004) pg/ml versus control 11596 (95% CI: 9083 to 14805) pg/ml, *P* = 0.02).

**Conclusion:**

ALM infusion induces a reversible hypotensive and hypometabolic state, attenuates tumor necrosis factor-α levels and improves cardiac and pulmonary function, and led to a transient drop in renal function that was reversed after the treatment was stopped.

## Introduction

Sepsis is associated with high mortality due to the development of cardiovascular dysfunction, lung injury and multiorgan failure [1,2]. The pathophysiology responsible for the poor outcomes of sepsis is believed to be associated with a simultaneous activation of pro-inflammatory and anti-inflammatory pathways with different phases during the course of sepsis dominated by either hyperinflammation or immunosuppression [3,4]. Initially the innate immune system is activated in response to microorganisms, leading to production of cytokines, reactive oxygen species, and activation of leukocytes [5,6].

The combination of adenosine and lidocaine (AL) is cardioprotective and is currently used as a cardioplegia strategy in cardiac surgery [7,8]. AL has also been shown to suppress neutrophil inflammatory functions to a greater extent than either drug alone [9]. The cardioprotective and anti-inflammatory properties of AL were expanded to a porcine model of cardiac arrest and resuscitation [10]. In addition, the combination of AL and Mg^2+^ (ALM) has been reported to improve cardiovascular, hemodynamic and pulmonary function and to reduce whole body oxygen consumption (VO_2_) following severe hemorrhagic shock and resuscitation [11-14]. Since cardiovascular dysfunction and respiratory failure are the most frequent causes of early death in septic patients [15], the effects of ALM may also be protective in the setting of sepsis and systemic inflammation. In support of this, the rat model of cecal ligation and puncture demonstrated that ALM prevented coagulopathy and reduced pulmonary edema while temporarily inducing reversible hypotension [16]. The current study tested the hypothesis that intervention with ALM will reduce tumor necrosis factor alpha (TNFα) peak levels and improve cardiovascular and pulmonary function in response to lipopolysaccharide in a porcine model.

## Materials and methods

The study was approved by the National Committee on Animal Research Ethics (2012-15-2934-00446; Glostrup, Denmark) and was conducted in accordance with the Principles of Laboratory Animal Care [17].

### Animal preparation

Sixteen female crossbred Landrace/Yorkshire/Duroc pigs (37 to 42 kg) were fasted overnight, but were allowed free access to water. Anesthesia was induced with midazolam (20 mg) and s-ketamin (250 mg) and maintained with fentanyl (60 μg∙kg^–1^∙hour^–1^) and midazolam (6 mg∙kg^–1^∙hour^–1^) as used in previous studies [13,18]. The animals were intubated and ventilated using pressure control ventilation with the volume guaranteed (S/5 Avance; Datex Ohmeda, Madison, WI, USA) at a positive end-expiratory pressure of 5 cmH_2_O, a fraction of inspired oxygen (FiO_2_) of 0.4, and a tidal volume of 10 ml/kg. The ventilation rate was adjusted to maintain arterial partial pressure of carbon dioxide between 41 and 45 mmHg. The body temperature was maintained around 38 to 38.5°C. All animals received a bolus of isotonic saline 10 ml/kg at baseline and a maintenance rate of 15 ml∙kg^–1^.hour^–1^ during lipopolysaccharide infusion.

### Surgical preparations and monitoring

Vascular sheaths were inserted into the carotid artery and both external jugular veins. A pressure–volume catheter (Transonic SciSense, London, Ontario, Canada) was inserted into the left ventricle through the right carotid artery. A pulmonary artery catheter (CCOmbo; Edwards Lifesciences, Irvine, CA, USA) was inserted into the pulmonary artery through the right external jugular vein to monitor cardiac output and the core temperature. A PTS® sizing balloon (NMT Medical, Boston MA, USA) was inserted in the left external jugular vein and positioned into the vena cava to occlude venous return during pressure–volume measurements. A bladder catheter was placed for urine collection.

Systemic vascular resistance (dyn.s/cm^5^) was calculated as:

$$ \mathrm{Systemic}\ \mathrm{vascular}\ \mathrm{resistance} = 80 \times \left(\mathrm{MAP}\ \hbox{--}\ \mathrm{central}\ \mathrm{venous}\ \mathrm{pressure}\right)/\mathrm{cardiac}\ \mathrm{output} $$

where MAP is the mean arterial pressure. Pulmonary vascular resistance (PVR, dyn.s/cm^5^) was calculated as:

$$ \mathrm{P}\mathrm{V}\mathrm{R} = 80 \times \left(\mathrm{MPAP}\ \hbox{--}\ \mathrm{pulmonary}\ \mathrm{capillary}\ \mathrm{wedge}\ \mathrm{pressure}\right)/\mathrm{cardiac}\ \mathrm{output} $$

where MPAP is the mean pulmonary arterial pressure.

### Experimental protocol

**Figure 1:**
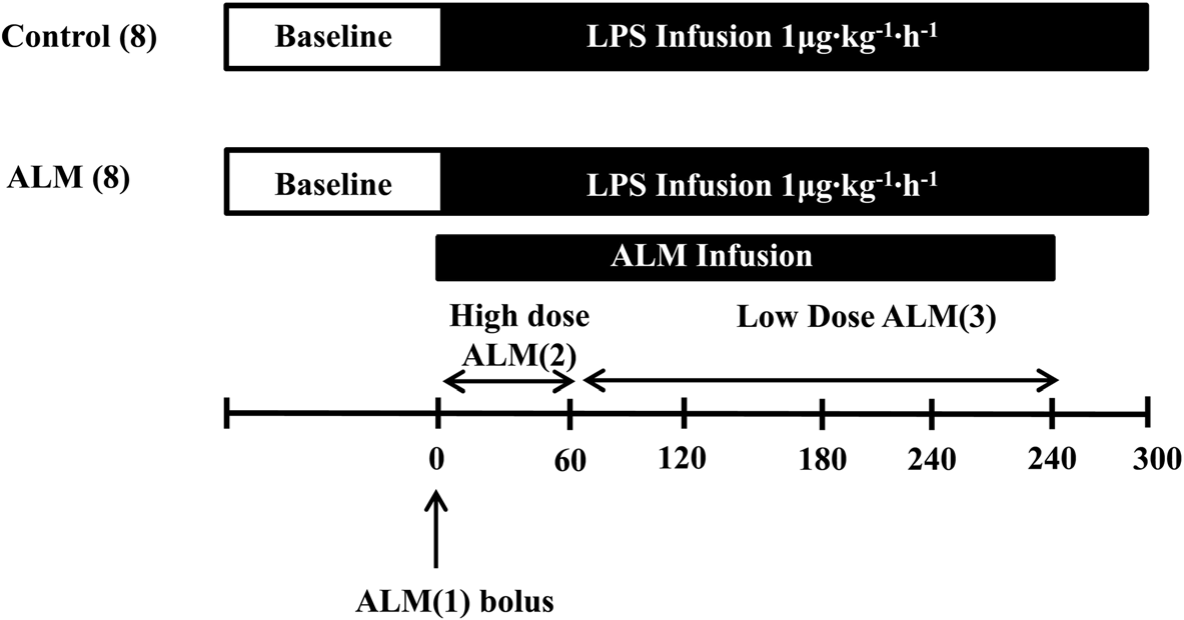
**Schematic diagram of the study protocol.** Pigs were randomly assigned in a blinded manner to one of two groups: Group 1, control (*n* = 8); Group 2, adenosine, lidocaine and magnesium (ALM; *n* = 8). Animals were subjected to endotoxemia by infusion of *Escherichia coli* lipopolysaccharide (LPS) at a rate of 1 μg∙kg^–1^.hour^–1^ for 5 hours. As LPS infusion was started, animals were loaded with a high-dose bolus infusion of ALM (ALM(1)) followed by a continuous infusion of ALM (ALM(2)) for 1 hour, after which the formulation was decreased (ALM(3)) to minimize hypotension.

After instrumentation, each animal, was randomly assigned to one of two groups: Group 1, control (*n* = 8); Group 2, ALM (*n* = 8) (Figure 1). Following surgery and instrumentation, randomization was performed by a laboratory technician drawing either control or ALM labels from a paper bag. The primary investigators were blinded to group assignments prior to infusion of ALM. With initiation of ALM infusion there was a significant drop in blood pressure, preventing blinding during the remainder of the study. Analysis of blood samples and data analysis were blinded to group assignment.

After randomization, endotoxemia was induced by infusion of *Escherichia coli* lipopolysaccharide (0111:B4, lot 011 m4008; Sigma-Aldrich, Broendby, Denmark) at a rate of 1 μg∙kg^–1^∙hour^–1^ for 5 hours [18]. In both groups, if MPAP increased to the level of MAP during the first hour of ALM infusion where MPAP levels are at the highest, an epinephrine bolus at a fixed dose of 0.002 mg was given to avoid circulatory collapse and death as reported in previous studies [18,19]. Therapy was guided by an increase in MAP and an increase in the difference between MPAP and MAP.

In the event of hypoxia (arterial partial pressure of oxygen (PaO_2_) < 12 kPa), FiO_2_ was increased to 0.60 initially, and then if inadequate to 0.80.

### Adenosine, lidocaine and magnesium treatment

Doses were determined by previous studies and pilot experiments using a three-tier ALM strategy (Figure 1) [13,20,21]. As lipopolysaccharide infusion was started, animals were loaded with a bolus infusion of ALM(1) (adenosine (0.82 mg/kg), lidocaine (1.76 mg/kg) and magnesium sulfate (0.92 mg/kg)) [10]; this was followed by a continuous infusion of ALM(2) using adenosine (300 μg∙kg^–1^∙minute^–1^), lidocaine (600 μg∙kg^–1^∙minute^–1^) and magnesium sulfate (336 μg∙kg^–1^∙minute^–1^) for 1 hour, after which the formulation was decreased to adenosine (240 μg∙kg^–1^∙minute^–1^), lidocaine (480 μg∙kg^–1^∙minute^–1^) and magnesium sulfate (268 μg∙kg^–1^∙minute^–1^) (ALM(3)) to minimize hypotension. For continuous infusion, drugs were dissolved in 1 l normal saline. In the control group, saline was used as a vehicle infusion and the rate of infusion was turned off after 4 hours. Observation was continued for a total of 5 hours.

### Oxygen consumption

VO_2_ was calculated as the product of the arterial–mixed venous oxygen content difference and cardiac output as described previously [13]. Oxygen delivery is calculated as the product of cardiac output and arterial oxygen content, while the oxygen extraction ratio is calculated as the ratio of arterial–venous difference and arterial oxygen content.

### Analysis of blood and urine samples

Arterial blood gas analysis was performed every half hour (ABL700; Radiometer, Broenshoej, Denmark). Blood plasma and urine samples were collected hourly. Blood samples were analyzed for creatinine, while urinary samples were analyzed for creatinine, protein and *N*-acetyl-β-d-glucosaminidase (NAGase) activity as described previously [13]. Urinary levels of neutrophil gelatinase-associated lipocalin (NGAL) were determined using a commercially available enzyme-linked immunosorbent assay kit (BioPorto Diagnostics A/S, Gentofte, Denmark) [22]. NGAL and NAGase are both markers of tubular injury. Intra-assay and inter-assay precisions were 2.71 and 6.27% respectively. NAGase activity, protein and NGAL concentrations in urine were divided by urinary creatinine concentrations to correct for urine output.

### Multiplex cytokine analysis

The concentration of the cytokines interleukin (IL)-6, IL-10, and TNFα were determined using a commercially available kit (Procarta® Porcine Cytokine Assay Kit; Panomics, San Diego, CA, USA) [18]. Detection limits were, 4.39 pg/ml for IL-6, 15.41 pg/ml for IL-10, and 14.45 pg/ml for TNFα. Inter-assay variations were 4 to 13%, and intra-assay variations were 1 to 5%.

### Leukocyte superoxide production

Blood samples were collected hourly and the number of leukocytes was quantified using an Automated Hematology Analyzer (KX-21 N; Sysmex Europe GmbH, Norderstedt, Germany). Leukocyte superoxide anion (˙O_2_^–^) generation was quantified using lucigenin-enhanced chemiluminescence [9]. Each whole blood sample was divided into two aliquots: whole blood alone; and whole blood + 0.2 mg/ml opsonized zymosan. The leukocyte superoxide anion component of the overall signal was demonstrated by adding superoxide dismutase (3 mg/ml; Sigma Chemicals, St. Louis, MO, USA). Lucigenin-enhanced chemiluminescence was recorded over 15 minutes in a Luminometer (Autolumat LP9507; Berthold Tech, Bad Wildbad, Germany) and expressed as relative light units per 10^6^ leukocytes. Data at different time points are expressed as a percentage of baseline chemiluminescence.

### Pulmonary function

The alveolar–arterial oxygen difference was calculated using the simplified alveolar gas equation:

$$ \mathrm{Pa}{\mathrm{O}}_2 = \left({\mathrm{P}}_{\mathrm{ATM}}\hbox{--}\ {\mathrm{P}}_{\mathrm{H}2\mathrm{O}}\right) \times \mathrm{F}\mathrm{i}{\mathrm{O}}_2\hbox{--}\ \mathrm{P}\mathrm{a}\mathrm{C}{\mathrm{O}}_2/\mathrm{R} $$

where P_ATM_ is the atmospheric pressure, P_H2O_ is the saturated vapor pressure of water (49.7 mmHg), PaCO_2_ is the arterial partial pressure of carbon dioxide and R is the respiratory quotient (0.8) [23]. To determine the wet/dry lung tissue weight ratio, representative samples of the right upper lung were weighed (wet weight) and placed in an oven at 70°C until there was no further weight loss (dry weight).

### Cardiac function

Real-time pressure–volume loops were obtained using the ADVantage™ system (Transonic SciSense), which uses an admittance catheter to simultaneously measure left ventricular pressure and admittance [24]. Data were continuously recorded using a multichannel acquisition system and Labchart software (ADInstruments, Oxford, UK). The following pressure-derived data were recorded: end systolic pressure, end diastolic pressure, time constant of isovolumic relaxation (Tau), maximum rate of pressure development over time (d*P*/d*t*_max_), and maximum rate of pressure decrease over time (d*P*/d*t*_min_). Preload was reduced by inflating the vena caval sizing catheter during respiratory apnea to obtain declining left ventricular pressure–volume loops from which the load-independent indices of contractility were calculated: preload recruitable stroke work (PRSW), end systolic pressure–volume relationship (end systolic elastance (Ees)), and end diastolic pressure–volume relationship. Arterial–ventricular coupling was described as the Arterial elastance (Ea) / Ees ratio. The optimal Ea/Ees ratio is approximately 1 and a deviation from this indicates a decrease in arterial–ventricular coupling efficiency and cardiac performance.

### Statistical analysis

For continuous variables, a repeated-measures analysis of variance was used to analyze data for time-dependent and between-group differences. It was determined *a priori* to perform *post-hoc* pairwise comparisons at baseline and at the end of the study; comparisons beyond this were adjusted for multiple compassions (Sidak). The repeated-measurements analysis of variance was *a priori* divided into analysis of: the entire study period, and the 4-hour ALM infusion period. The assumptions of the models were investigated by inspecting scatter plots of the residuals versus fitted values, and normal quantile plots of the residuals and data were logarithmically transformed when necessary. If data did not fulfill assumptions for repeated-measures analysis of variance despite logarithmical transformation, they were analyzed using multivariate repeated-measurements analysis of variance as reported previously [14,18].

All variables are presented on the original scale of measurement as mean/median and 95% confidence intervals (CIs). Two-tailed *P* < 0.05 was considered statistically significant.

Eight pigs being included in each group was based on power calculations with data from six separate pilot studies with respect to peak TNFα levels at 90 minutes and a change in VO_2_ from before/after infusion was discontinued (TNFα: difference = 3,353 pg/ml; standard deviation = 1,480; α = 0.05 and β =0.1, *n* = 5: VO_2_: difference = 79 ml oxygen/minute; standard deviation control = 54/ALM = 29; α = 0.05 and β = 0.1, *n* = 7). Power calculations were performed with the primary endpoint TNFα and the secondary endpoint VO_2_ since we wanted to investigate whether the known anti-inflammatory and metabolic lowering effects of ALM would translate into an improvement in organ function. The analyses were performed using Stata 12.1 (StataCorp LP, College Station, TX, USA).

## Results

### Hemodynamic function

**Figure 2:**
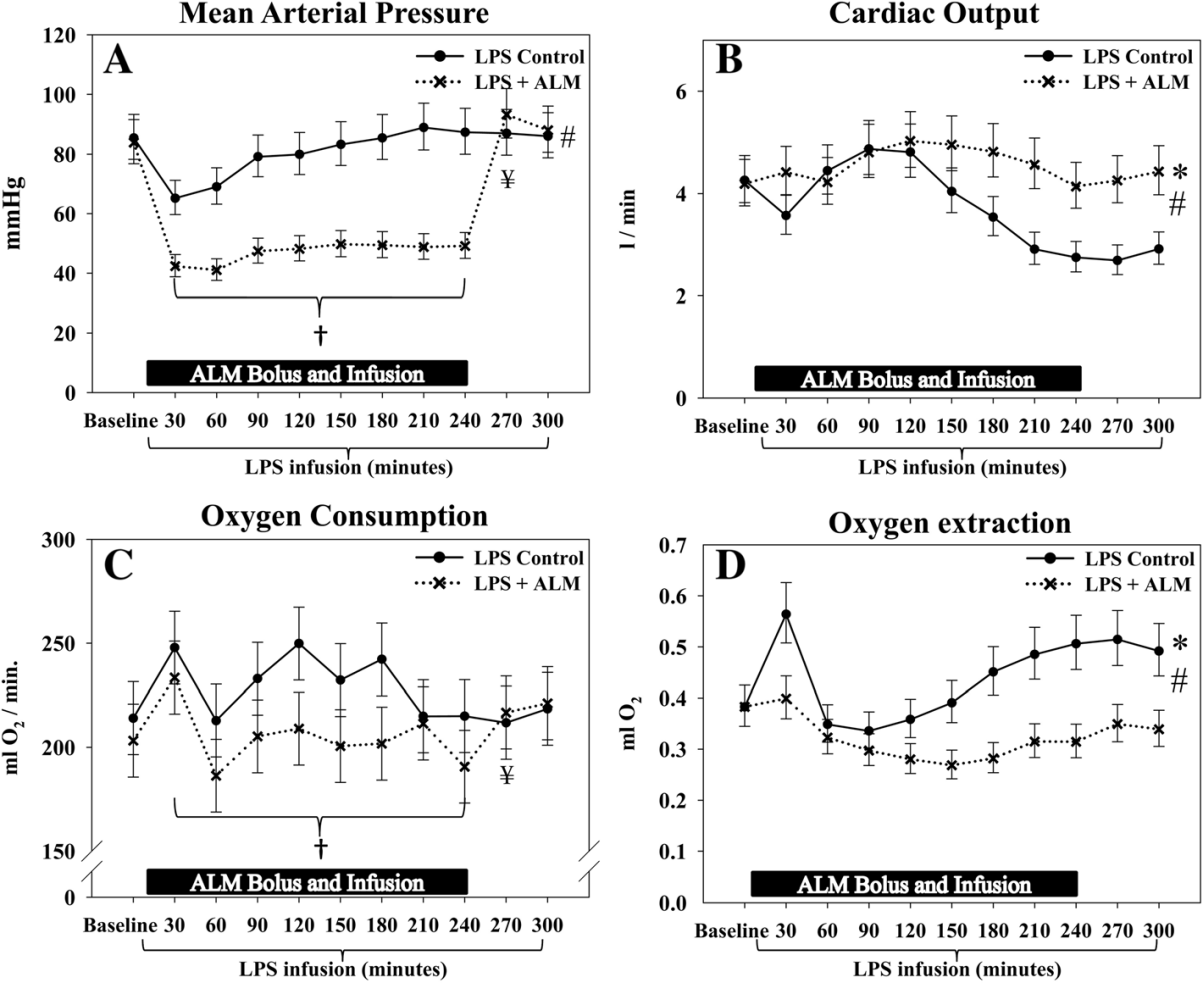
**Hemodynamic and metabolic data.** Treatment with adenosine, lidocaine and magnesium (ALM) induced reversible hypotension, increased cardiac output and decreased oxygen consumption and oxygen extraction during infusion of ALM. **(A)** Mean arterial pressure. **(B)** Cardiac output. **(C)** Whole body oxygen consumption. **(D)** Whole body oxygen extraction. *Significant difference at the end of the study. #Significantly different change over time between groups. †Significantly different mean/median level during infusion of ALM. ¥Significant difference before/after cessation of ALM infusion. Data presented as median (95% confidence interval), except for oxygen consumption which is presented as mean (95% confidence interval). LPS, lipopolysaccharide.

All reported baseline values are prior to the start of lipopolysaccharide and ALM infusion. ALM infusion resulted in a significantly lower MAP during the 4-hour treatment period (Figure 2A). At the end of ALM infusion, MAP immediately returned to control group values. The lower MAP during infusion of ALM was due to a lower systemic vascular resistance (Table 1) despite a significantly higher cardiac output (Figure 2B).

**Table 1:** **Systemic hemodynamic variables**

	**Baseline**	**30 minutes**	**60 minutes**	**90 minutes**	**120 minutes**	**150 minutes**	**180 minutes**	**210 minutes**	**240 minutes**	**270 minutes**	**300 minutes**
Heart rate (min^–1^)											
Control	69 (62 to 76)	83 (76 to 90)	84 (77 to 91)	74 (67 to 81)	73 (66 to 80)	77 (69 to 84)	75 (68 to 82)	70 (63 to 77)	69 (62 to 76)	70 (63 to 77)	71 (64 to 78)
ALM^a^	69 (62 to 76)	70 (63 to 77)	72 (65 to 79)	76 (69 to 83)	81 (74 to 88)	84 (77 to 91)	84 (77 to 91)	81 (74 to 88)	80 (73 to 87)	84 (76 to 91)	84^b^ (77 to 91)
Systemic vascular resistance (dyn.s/cm^5^)											
Control	1,526 (1,328 to 1,753)	1,242 (1,081 to 1,427)	1,141 (993 to 1,310)	1,177 (1,024 to 1,352)	1,210 (1,054 to 1,390)	1,501 (1,306 to 1,724)	1,768 (1,539 to 2,031)	2,245 (1,954 to 2,579)	2,327 (2,025 to 2,673)	2,357 (2,0151 to 2,707)	2,145 (1,867 to 2,464)
ALM^a^	1,500 (1,306 to 1,723)	607 (528 to 697)	63 (553 to 730)	652 (568 to 749)	644 (561 to 740)	689 (600 to 792)	710 (618 to 816)	742 (646 to 852)	816 (710 to 937)	1,630^c^ (1,418 to 1,872)	1,472^b^ (1,282 to 1,691)
Pulmonary vascular resistance (dyn. s/cm^5^)											
Control	131 (109 to 157)	688 (574 to 826)	461 (384 to 553)	331 (276 to 397)	318 (265 to 382)	481 (401 to 577)	585 (488 to 702)	656 (547 to 787)	665 (554 to 797)	640 (533 to 767)	567 (473 to 681)
ALM^a^	154 (129 to 185)	173^d^ (144 to 208)	165 (138 to 198)	190 (159 to 228)	246 (205 to 295)	314 (262 to 377)	324 (270 to 388)	333 (277 to 399)	330 (276 to 396)	351 (293 to 421)	300^b^ (250 to 360)
Stroke volume (ml)											
Control	63 (58 to 68)	46 (41 to 50)	54 (49 to 59)	67 (62 to 72)	67 (62 to 72)	54 (49 to 59)	48 (43 to 53)	43 (38 to 48)	40 (35 to 45)	39 (34 to 44)	42 (37 to 47)
ALM^a^	61 (56 to 66)	65 (61 to 70)	60 (55 to 65)	65 (60 to 69)	63 (58 to 68)	59 (55 to 64)	58 (54 to 63)	57 (52 to 62)	52 (48 to 57)	52 (47 to 57)	53^b^ (48 to 58)

Data presented as median (95% confidence interval), except for heart rate and stroke volume which are presented as mean (95% confidence interval). ALM, adenosine, lidocaine and magnesium. ^a^Significantly different change over time between groups. ^b^Significant difference at the end of the study. ^c^Significant difference before/after cessation ALM infusion. ^d^Significant difference between groups (Sidak).

At the end of the study, both the heart rate and stroke volume (SV) were significantly higher in the ALM group versus the control group (Table 1). The use of intravenous epinephrine was protocol driven to avoid circulatory collapse and death if MPAP was equal to or greater than MAP during the first 60 minutes [18]. A significantly lower cumulative dose of epinephrine was administered according to this protocol in the ALM group (ALM median, 0 (range 0 to 0.2) μg vs. control median, 0.6 (range 0 to 2.4) μg, *P* = 0.025).

### Inflammation

**Figure 3:**
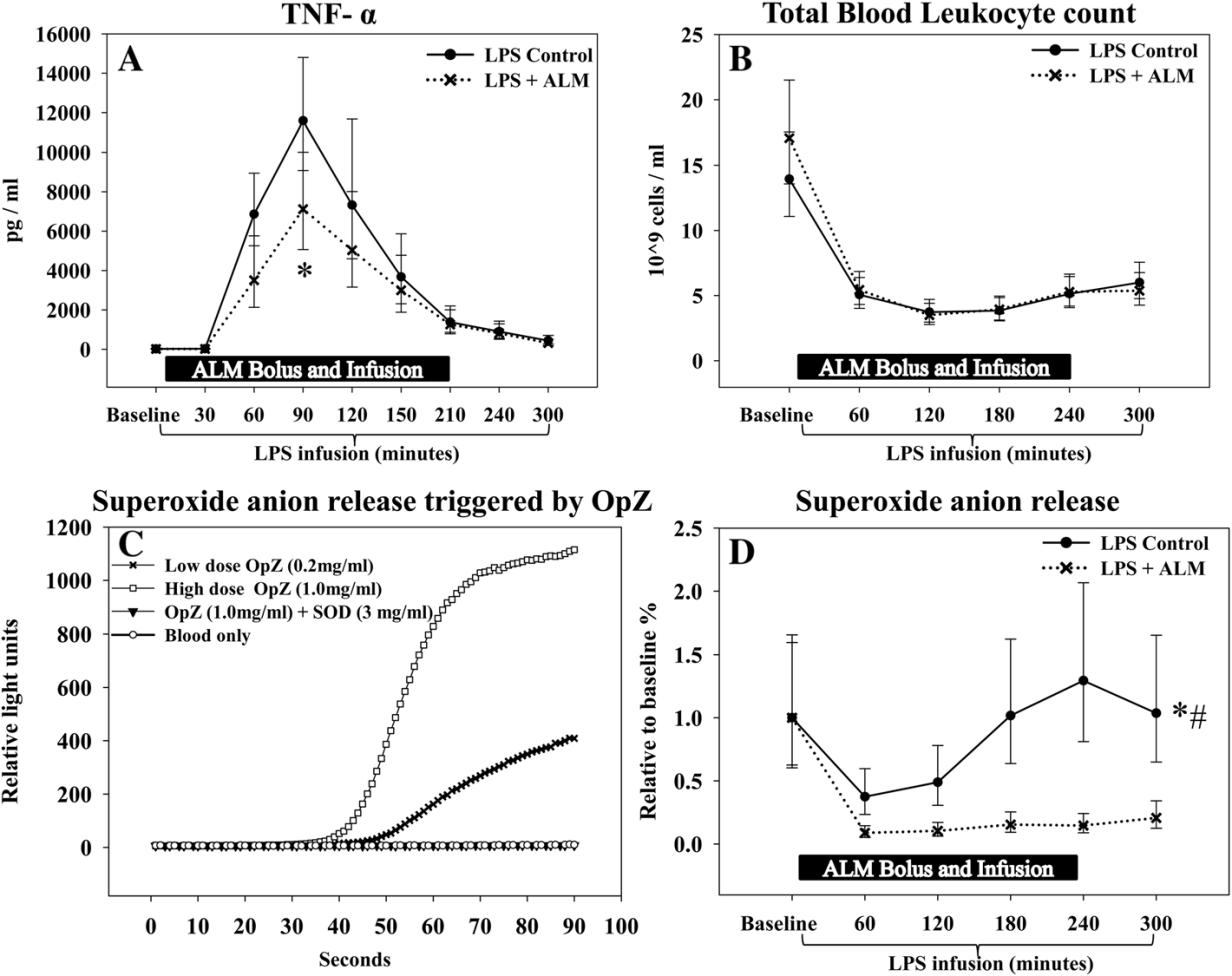
**Tumor necrosis factor alpha and leukocyte data. (A)** Peak tumor necrosis factor alpha (TNFα) levels were significantly lower in the treatment group. **(B)** Total blood leukocyte count decreased over time with no group difference. **(C)** A dose–response relationship with regards to superoxide anion production was observed and the leukocyte superoxide anion (˙O_2_^–^) component of the overall signal was demonstrated by adding superoxide dismutase. **(D)** Superoxide anion production, stimulated by low-dose opsonized zymosan 0.02 mg/ml, was significantly attenuated in the treatment group. *Significant difference between groups. #Significantly different change over time between groups. Data presented as median (95% confidence interval). ALM, adenosine, lidocaine and magnesium; LPS, lipopolysaccharide; OpZ, opsonized zymosan; SOD, superoxide dismutase.

Infusion of lipopolysaccharide caused a characteristic increase in plasma cytokines (Table 2, Figure 3A). Peak TNFα levels after 90 minutes of lipopolysaccharide infusion were significantly lower in the ALM group (control/ALM ratio, 1.63 (95% CI: 1.11 to 2.38); *P* = 0.02). No significant difference existed between groups with regards to IL-6 or IL-10. The total blood leukocyte count decreased over time, with no group differences (Figure 3B). *In vitro* superoxide anion production was significantly lower in the ALM group when compared with the control group (Figure 3C,D).

**Table 2:** **Plasma cytokines and renal function**

	**Baseline**	**30 minutes**	**60 minutes**	**90 minutes**	**120 minutes**	**150 minutes**	**180 minutes**	**210 minutes**	**240 minutes**	**300 minutes**
Interleukin-6 (pg/ml)										
Control	6 (4 to 10)	5 (3 to 8)	4 (2 to 6)	32 (19 to 53)	107 (65 to 178)	168 (102 to 279)		221 (133 to 366)	174 (105 to 289)	83 (50 to 138)
ALM	4 (3 to 7)	4 (3 to 7)	6 (4 to 10)	45 (27 to 75)	177 (107 to 293)	272 (164 to 451)		339 (204 to 561)	266 (161 to 441)	90 (54 to 149)
Interleukin-10 (pg/ml)										
Control	5 (3 to 8)	10 (6 to 17)	327 (201 to 532)	391 (240 to 636)	215 (132 to 350)	213 (131 to 347)		392 (241 to 638)	419 (257 to 681)	315 (194 to 512)
ALM	6 (4 to 11)	14 (8 to 22)	303 (186 to 492)	463 (285 to 754)	341 (209 to 554)	297 (182 to 483)		347 (213 to 564)	354 (218 to 576)	383 (235 to 623)
Urinary protein/creatinine ratio (μg/μmol)										
Control	7.5 (5.5 to 10.3)		7.6 (5.5 to 10.4)		9.4 (6.8 to 12.9)		10.1 (7.4 to 13.9)		10.2 (7.5 to 14.1)	11.1 (8.1 to 15.2)
ALM^a^	9.1 (6.6 to 12.5)		8.4 (6.1 to 11.5)		14.1 (10.1 to 19.3)		24.3 (17.7 to 33.4)		19.5 (14.2 to 26.7)	14.7 (10.7 to 20.2)
Urinary NAGase/creatinine ratio (U/mmol)										
Control	2.2 (1.5 to 3.3)		2.0 (1.3 to 3.0)		2.2 (1.5 to 3.3)		1.8 (1.2 to 2.8)		1.8 (1.2 to 2.8)	2.2 (1.4 to 3.3)
ALM^b^	2.1 (1.4 to 3.2)		2.1 (1.4 to 3.2)		3.0 (2.0 to 4.5)		6.3 (4.2 to 9.5)		6.5 (4.3 to 9.7)	3.0^c^ (2.0 to 4.5)

Data presented as median (95% confidence interval). ALM, adenosine, lidocaine and magnesium; NAGase, *N*-acetyl-β-d-glucosaminidase. ^a^Significantly different mean/median level. ^b^Significantly different change over time between groups. ^c^Significant difference before/after cessation ALM infusion.

### Metabolic function

As a consequence of the higher cardiac output, global oxygen delivery was significantly greater in the ALM group (Table 3). However, the average whole body VO_2_ during the infusion period was significantly lower than for controls (ALM, 205 (95% CI: 192 to 217) ml oxygen/minute vs. control, 231 (95% CI: 219 to 243) ml oxygen/minute, *P* = 0.016; Figure 2C), while it immediately returned to control group values after cessation of ALM treatment.

**Table 3:** **Oxygen consumption variables**

	**Baseline**	**30 minutes**	**60 minutes**	**90 minutes**	**120 minutes**	**150 minutes**	**180 minutes**	**210 minutes**	**240 minutes**	**270 minutes**	**300 minutes**
Oxygen delivery (ml O_2_/minute)											
Control	556 (492 to 629)	436 (386 to 493)	607 (537 to 686)	692 (612 to 782)	696 (616 to 787)	592 (523 to 669)	533 (472 to 603)	438 (388 to 495)	423 (374 to 478)	410 (363 to 463)	441 (390 to 498)
ALM^a^	527 (466 to 595)	569 (504 to 644)	571 (505 to 645)	686 (607 to 776)	742 (656 to 838)	740 (655 to 837)	710 (628 to 803)	670 (592 to 757)	602 (533 to 681)	619 (548 to 700)	648^b^ (574 to 733)
Arterial–venous difference (ml O_2_/l blood)											
Control	50 (46 to 55)	69 (63 to 75)	48 (44 to 52)	48 (44 to 52)	52 (47 to 57)	57 (52 to 63)	68 (62 to 74)	73 (67 to 80)	78 (71 to 85)	78 (72 to 86)	74 (68 to 81)
ALM^a^	48 (44 to 53)	51 (47 to 56)	44 (40 to 48)	42 (39 to 46)	41 (38 to 45)	40 (37 to 44)	42 (38 to 45)	46 (42 to 51)	46 (42 to 50)	51 (46 to 56)	50^b^ (45 to 54)
Respiratory rate (min^–1^)											
Control	12 (11 to 13)	12 (11 to 12)	13 (12 to 13)	13 (12 to 14)	13 (13 to 14)	13 (13 to 14)	14 (13 to 14)	14 (13 to 15)	14 (13 to 15)	14 (14 to 15)	14 (14 to 15)
ALM	13 (13 to 14)	13 (12 to 13)	13 (12 to 13)	13 (12 to 14)	13 (12 to 14)	14 (13 to 14)	13 (13 to 14)	14 (13 to 14)	14 (13 to 14)	14 (13 to 15)	14 (14 to 15)
Airway peak pressure (cmH_2_O)											
Control	19 (18 to 20)	21 (20 to 22)	21 (20 to 22)	21 (20 to 22)	22 (21 to 23)	23 (22 to 24)	24 (23 to 25)	24 (23 to 25)	25 (24 to 26)	25 (24 to 26)	25 (24 to 26)
ALM	19 (18 to 20)	21 (20 to 22)	21 (20 to 22)	22 (21 to 23)	22 (21 to 23)	23 (22 to 24)	24 (22 to 25)	24 (23 to 25)	24 (23 to 25)	24 (23 to 25)	24 (23 to 25)

Data presented as median (95% confidence interval), except for temperature which is presented as mean (95% confidence interval). ALM, adenosine, lidocaine and magnesium. ^a^Significant different change over time between groups. ^b^Significant difference at the end of the study.

The oxygen extraction ratio was unchanged in the ALM group, supporting a favorable oxygen supply/demand status (Figure 2D). In direct contrast, the ratio increased over time in the control group, consistent with inadequate delivery of oxygen.

Lactate was significantly lower in the ALM group at the end of the study (Table 4).

**Table 4:** **Systemic arterial gas and metabolic variables**

	**Baseline**	**30 minutes**	**60 minutes**	**90 minutes**	**120 minutes**	**150 minutes**	**180 minutes**	**210 minutes**	**240 minutes**	**270 minutes**	**300 minutes**
Arterial pH											
Control	7.48 (7.47 to 7.49)	7.48 (7.47 to 7.50)	7.44 (7.43 to 7.46)	7.44 (7.42 to 7.45)	7.44 (7.42 to 7.45)	7.42 (7.40 to 7.43)	7.41 (7.40 to 7.43)	7.41 (7.39 to 7.42)	7.41 (7.40 to 7.43)	7.41 (7.40 to 7.42)	7.41 (7.39 to 7.42)
ALM	7.48 (7.47 to 7.50)	7.48 (7.46 to 7.49)	7.45 (7.44 to 7.46)	7.44 (7.42 to 7.45)	7.41 (7.40 to 7.43)	7.41 (7.40 to 7.43)	7.40 (7.39 to 7.42)	7.40 (7.39 to 7.42)	7.40 (7.38 to 7.42)	7.40 (7.38 to 7.41)	7.40 (7.39 to 7.41)
PaO_2_ (mmHg)											
Control	182 (161 to 205)	118 (105 to 135)	154 (136 to 173)	157 (139 to 177)	144 (127 to 162)	117 (103 to 132)	111 (98 to 125)	110 (98 to 124)	127 (112 to 143)	126 (111 to 142)	115 (102 to 130)
ALM^a^	184 (163 to 207)	173 (154 to 196)	174 (154 to 197)	169 (150 to 191)	168 (148 to 189)	170 (150 to 191)	167 (148 to 188)	163 (144 to 184)	150 (133 to 169)	151 (134 to 171)	147^b^ (130 to 166)
PaCO_2_ (mmHg)											
Control	43 (42 to 44)	41 (40 to 42)	44 (42 to 45)	44 (42 to 45)	44 (43 to 45)	44 (43 to 46)	45 (44 to 46)	45 (44 to 46)	44 (43 to 45)	43 (42 to 44)	44 (43 to 45)
ALM	43 (41 to 44)	41 (40 to 42)	42 (41 to 44)	42 (41 to 44)	44 (43 to 35)	44 (42 to 45)	44 (43 to 45)	43 (42 to 44)	44 (42 to 45)	45 (44 to 46)	45 (44 to 46)
ETCO_2_ (mmHg)											
Control	43 (42 to 45)	42 (41 to 43)	45 (44 to 47)	45 (44 to 46)	44 (43 to 45)	43 (42 to 45)	42 (40 to 43)	41 (39 to 42)	40 v(39 to 41)	40 (39 to 41)	40 (39 to 41)
ALM^c^	45 (44 to 46)	42 (41 to 44)	45 (44 to 46)	45 (43 to 46)	45 (44 to 47)	44 (43 to 46)	45 (44 to 46)	44 (42 to 45)	44 (43 to 45)	44 (43 to 45)	44^b^ (42 to 45)
HCO_3_^–^ (mmol/l)											
Control	31.4 (30.6 to 32.2)	30.5 (29.7 to 31.3)	29.1 (28.3 to 29.9)	28.7 (27.9 to 29.4)	28.7 (27.9 to 29.4)	27.8 (27.1 to 28.6)	27.6 (26.8 to 28.4)	27.1 (26.3 to 27.9)	26.9 (26.1 to 27.7)	26.6 (25.8 to 27.4)	26.8 (26.0 to 27.6)
ALM	31.4 (30.7 to 32.2)	30.3 (29.5 to 31.0)	29.0 (28.2 to 29.8)	28.1 (27.3 to 28.8)	27.4 (26.6 to 28.1)	27.0 (26.2 to 27.7)	26.6 (25.8 to 27.4)	26.5 (25.7 to 27.3)	26.1 (25.3 to 26.9)	26.4 (25.6 to 27.1)	26.6 (25.8 to 27.4)
Lactate (mmol/l)											
Control	0.8 (0.6 to 0.9)	0.8 (0.6 to 1.0)	1.1 (0.9 to 1.2)	1.1 (0.9 to 1.2)	1.2 (1.0 to 1.3)	1.2 (1.1 to 1.4)	1.3 (1.1 to 1.4)	1.3 (1.2 to 1.5)	1.3 (1.2 to 1.5)	1.2 (1.1 to 1.4)	1.1 (0.9 to 1.2)
ALM^c^	0.7 (0.6 to 0.9)	1.0 (0.8 to 1.1)	1.2 (1.0 to 1.3)	1.2 (1.0 to 1.4)	1.2 (1.0 to 1.4)	1.2 (1.0 to 1.3)	1.2 (1.0 to 1.3)	1.1 (1.0 to 1.3)	1.1 (0.9 to 1.3)	1.0 (0.8 to 1.1)	0.8^b^ (0.7 to 1.0)

Data presented as median (95% confidence interval), except for pH, lactate, and ETCO_2_ which are presented as mean (95% confidence interval). ALM, adenosine, lidocaine and magnesium; ETCO_2_, end-tidal carbon dioxide; PaCO_2_, arterial partial pressure of carbon dioxide; PaO_2_, arterial partial pressure of oxygen. ^a^Significantly different mean/median level between groups. ^b^Significant difference at the end of the study. ^c^Significantly different change over time between groups.

### Pulmonary function

**Figure 4:**
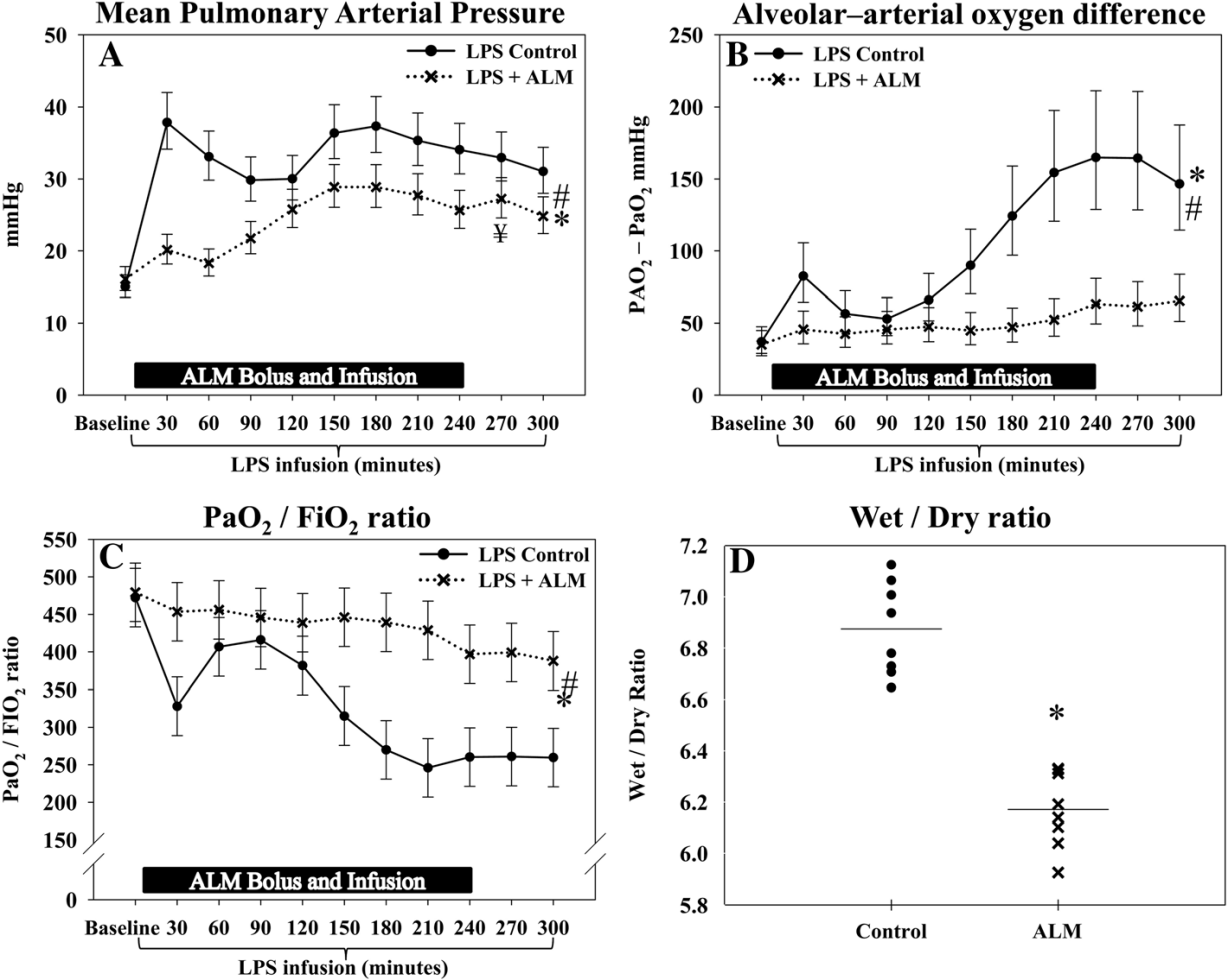
**Pulmonary function.** Infusion of adenosine, lidocaine and magnesium (ALM) improved pulmonary function, indicated by a lower mean pulmonary arterial pressure, a lower alveolar–arterial oxygen difference, a lower wet/dry ratio and a higher PaO_2_/FiO_2_ ratio. **(A)** Mean pulmonary arterial pressure. **(B)** Alveolar–arterial oxygen difference. **(C)** PaO_2_/FiO_2_ ratio. **(D)** Pulmonary wet/dry ratio. *Significant difference at the end of the study. #Significantly different change over time between groups. ¥Significant difference before/after cessation ALM infusion. Mean pulmonary arterial pressure and PAO_2_–PaO_2_ difference are presented as median (95% confidence interval), while the PaO_2_/FiO_2_ ratio is presented as mean (95% confidence interval). Pulmonary wet/dry ratio presented as mean and individual values. FiO_2_, inspired fraction of oxygen; LPS, lipopolysaccharide; PaO_2_, arterial partial pressure of oxygen; PAO_2_, alveolar partial pressure of oxygen.

Infusion of lipopolysaccharide caused a characteristic increase in MPAP with a peak at 30 minutes; this increase was avoided in the ALM group (Figure 4A). ALM maintained a significantly lower MPAP during the entire study. There was an initial peak in PVR at 30 minutes in the control group but this was not seen in the ALM group (Table 1). PVR continued to be lower during the entire study in the ALM group.

A positive end-expiratory pressure of 5 cmH_2_O and a tidal volume of 10 ml/kg were delivered to all pigs throughout the study. Peak respiratory pressures and respiratory rates increased over time in both groups with no difference between groups (Table 3).

The alveolar–arterial oxygen difference was maintained in the ALM group while it increased over time in the control group with a significant difference at the end of the study (Figure 4B). Similarly, the PaO_2_/FiO_2_ ratio was maintained in the ALM group, while it decreased over time in the control group, and ended at a significantly higher level in the ALM group (Figure 4C). Treatment with ALM significantly reduced the mean pulmonary wet/dry ratio when compared with the control group (Figure 4D).

### Cardiac function

**Figure 5:**
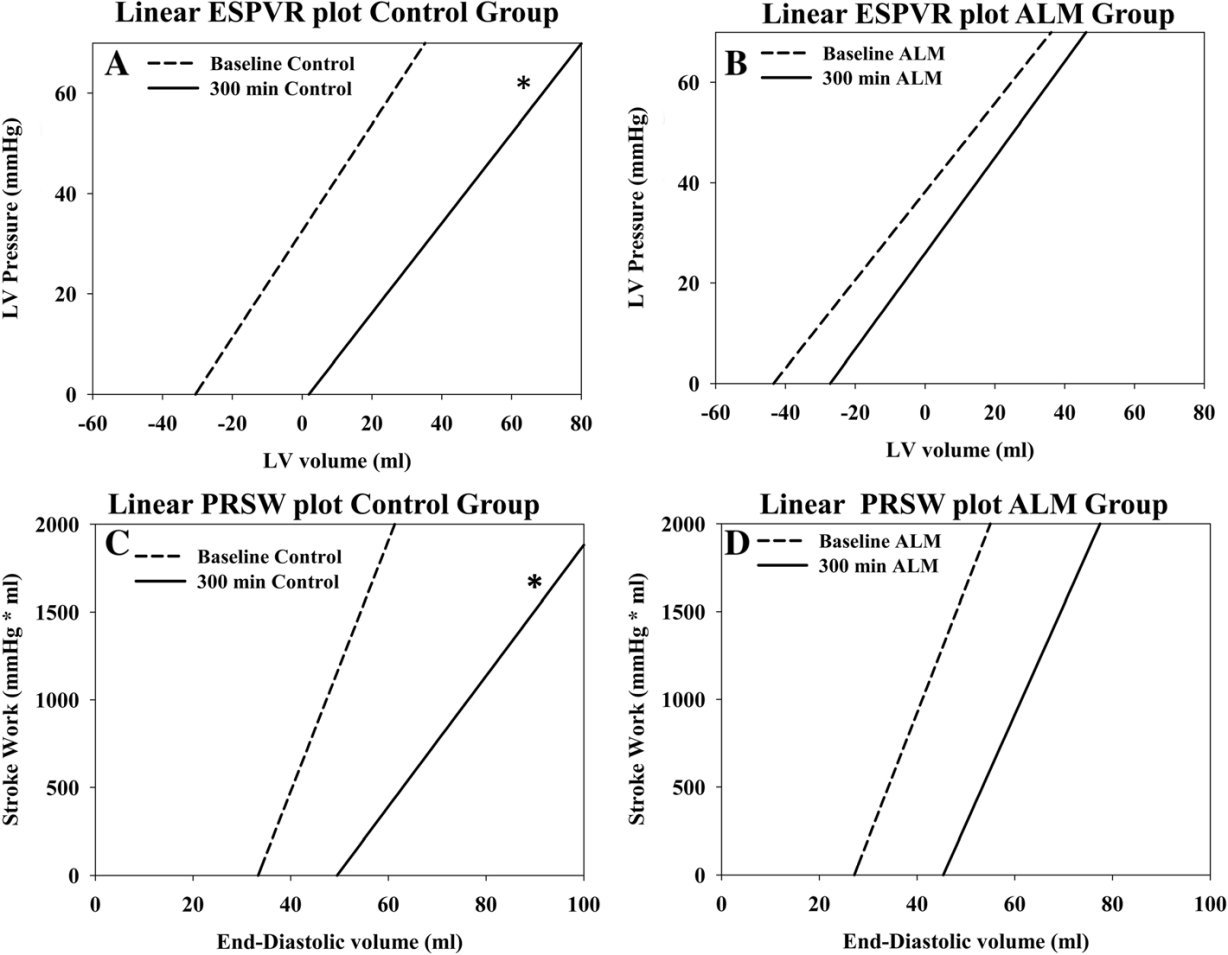
**Pressure–volume data.** Linear plots of the end systolic pressure–volume relationship (ESVPR; end systolic elastance) and the preload recruitable stroke work (PRSW) in the control group and the adenosine, lidocaine and magnesium (ALM) group at baseline and at the end of the study. **(A)** Linear plot of ESVPR in the control group at baseline and at the end of the study. **(B)** Linear plot of ESPVR in the adenosine, lidocaine and magnesium (ALM) group at baseline and at the end of the study. **(C)** Linear plot of PRSW in the control group at baseline and at the end of the study. **(D)** Linear plot of PRSW in the ALM group at baseline and at the end of the study. Both the end-systolic pressure–volume relationship and the preload recruitable stroke work are defined by a slope and a volume axis intercept taken from data presented in Table 4 and are presented since changes in the slope and intercept can be viewed simultaneously. *Significant difference at the end of the study. LV, left ventricular.

**Figure 6:**
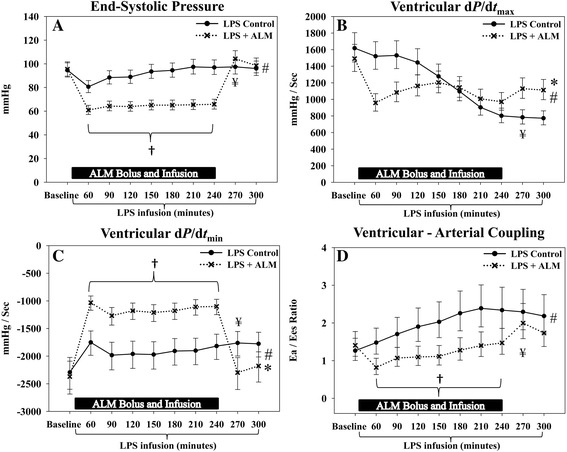
**Cardiac function.** During infusion of adenosine, lidocaine and magnesium (ALM), the end systolic pressure was significantly lower resulting in an improved arterial–ventricular coupling. Furthermore, systolic and diastolic function evaluated by d*P*/d*t*_max_ and d*P*/d*t*_min_ respectively was improved at the end of the study in the ALM group. **(A)** End systolic pressure. **(B)** Maximum rate of pressure development over time (d*P*/d*t*_max_). **(C)** Maximum rate of pressure decrease over time (d*P*/d*t*_min_). **(D)** Ventricular–arterial coupling. *Significant difference at the end of the study. #Significant different change over time between groups. †Significant different mean/median level during infusion of ALM. ¥Significant difference before/after cessation of ALM infusion. Data presented as median (95% confidence interval). Ea, arterial elastance; Ees, end systolic elastance (end systolic pressure–volume relationship); LPS, lipopolysaccharide.

The slope of Ees did not change significantly over time in either group (Figure 5A,B, Table 5). However, a rightward shift of the volume axis intercept was observed in the control group, consistent with a decrease in contractility; this shift was prevented in the ALM group (Figure 5A,B, Table 5). The slope of the PRSW, an index of overall cardiac performance, decreased in the control group but was preserved in the ALM group (Figure 5C,D, Table 5). In both groups there was a rightward shift in the intercept of PRSW with no significant group difference at the end of the study. Another index of cardiac contractility, d*P/*d*t*_max_ was significantly higher in the ALM group at the end of the study when compared with the control group, at equal pressures (Figure 6A,B). The end-diastolic pressure–volume relationship did not change significantly over time and there was no group difference (data not shown). However, diastolic function evaluated by d*P/*d*t*_min_ and Tau was significantly improved in the ALM group (Figure 6C, Table 5). Arterial–ventricular coupling (Ea/Ees) increased progressively in the controls during the course of the experiment, consistent with mismatched coupling. This was not observed in the ALM group during ALM infusion, whereas the Ea/Ees ratio increased to control group levels after infusion was discontinued (Figure 6D).

**Table 5:** **Cardiac function variables**

	**Baseline**	**60 minutes**	**90 minutes**	**120 minutes**	**150 minutes**	**180 minutes**	**210 minutes**	**240 minutes**	**270 minutes**	**300 minutes**
EEs slope (mmHg/ml)										
Control	1.01 (0.82 to 1.24)	0.88 (0.71 to 1.08)	0.69 (0.56 to 0.85)	0.64 (0.52 to 0.79)	0.73 (0.59 to 0.90)	0.72 (0.58 to 0.88)	0.81 (0.66 to 0.99)	0.89 (0.72 to 1.09)	0.94 (0.77 to 1.16)	0.88 (0.71 to 1.08)
ALM	0.86 (0.7 to 1.06)	1.05 (0.82 to 1.34)	0.79 (0.64 to 0.97)	0.79 (0.65 to 0.98)	0.81 (0.66 to 1.00)	0.74 (0.60 to 0.91)	0.78 (0.63 to 0.95)	0.73 (0.60 to 0.90)	0.82 (0.67 to 1.01)	0.89 (0.72 to 1.10)
EEs V_0_ (ml)										
Control	–38 (–56 to –20)	–40 (–58 to –22)	–64 (–82 to –45)	–63 (–81 to –45)	–51 (–69 to –32)	–47 (–65 to –28)	–25 (–43 to –7)	–10 (–28 to 9)	–5 (–23 to 13)	0 (–18 to 18)
ALM^a^	–48 (–66 to –30)	–7 (–29 to 14)	–25 (–43 to –7)	–26 (–45 to –8)	–29 (–47 to –11)	–31 (–49 to –13)	–23 (–41 to –5)	–30 (–48 to –12)	–39 (–57 to –21)	–33^b^ (–51 to –14)
PRSW slope (mmHg.ml/ml)										
Control	70 (58 to 84)	50 (41 to 60)	51 (42 to 61)	42 (35 to 51)	43 (36 to 52)	38 (32 to 46)	40 (33 to 48)	33 (27 to 39)	34 (28 to 41)	36 (30 to 43)
ALM^a^	70 (59 to 85)	72 (59 to 88)	64 (54 to 77)	61 (51 to 74)	57 (48 to 69)	56 (46 to 67)	48 (40 to 57)	58 (48 to 70)	66 (54 to 79)	61^b^ (51 to 74)
PRSW V_0_ (ml)										
Control	33 (22 to 44)	24 (13 to 34)	26 (15 to 37)	10 (–1 to 21)	23 (12 to 34)	25 (14 to 35)	45 (34 to 56)	41 (30 to 52)	51 (40 to 61)	51 (40 to 61)
ALM^a^	27 (16 to 38)	43 (31 to 56)	46 (35 to 57)	43 (32 to 53)	38 (27 to 48)	41 (30 to 52)	43 (32 to 54)	52 (41 to 62)	37^c^ (26 to 48)	45 (34 to 56)
Tau (milliseconds)										
Control	32 (29 to 35)	30 (28 to 33)	35 (32 to 38)	35 (32 to 38)	37 (34 to 40)	38 (35 to 42)	41 (38 to 45)	44 (40 to 48)	44 (41 to 48)	44 (40 to 48)
ALM^a^	31 (28 to 34)	33 (30 to 36)	31 (28 to 34)	30 (28 to 33)	32 (29 to 35)	33 (30 to 36)	35 (32 to 38)	36 (33 to 39)	37 (34 to 40)	36^b^ (33 to 39)
Aortic elastance (mmHg/ml)										
Control	1.3 (1.1 to 1.5)	1.3 (1.1 to 1.5)	1.2 (1.0 to 1.3)	1.2 (1.1 to 1.4)	1.5 (1.3 to 1.7)	1.6 (1.4 to 1.9)	1.9 (1.7 to 2.2)	2.1 (1.8 to 2.4)	2.2 (1.9 to 2.5)	1.9 (1.7 to 2.2)
ALM^a^	1.2 (1.1 to 1.4)	0.9 (0.8 to 1.0)	0.8 (0.7 to 1.0)	0.9 (0.8 to 1.0)	0.9 (0.8 to 1.0)	0.9 (0.8 to 1.1)	1.1 (0.9 to 1.2)	1.1 (0.9 to 1.2)	1.6^c^ (1.4 to 1.9)	1.5^b^ (1.3 to 1.8)

Data presented as median (95% confidence interval), except for Ees V_0_ and PRSW V_0_ which are presented as mean (95% confidence interval). ALM, adenosine, lidocaine and magnesium; Ees, end systolic elastance; PRSW, preload recruitable stroke work; V_0_, volume axis intercept. ^a^Significantly different change over time between groups. ^b^Significant difference at the end of the study. ^c^Significant difference before/after cessation ALM infusion.

### Renal function

**Figure 7:**
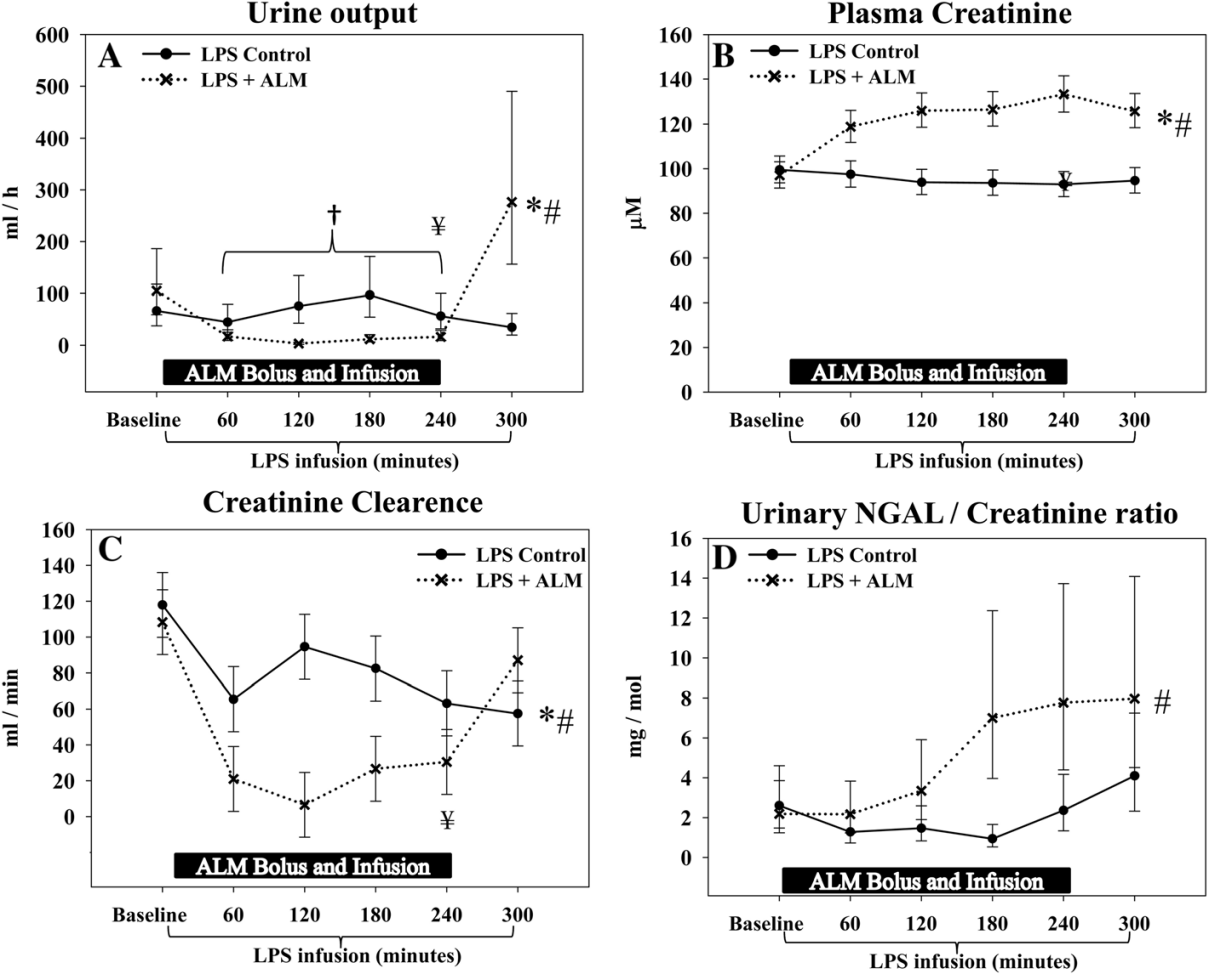
**Renal function.** A temporary impairment of renal function was observed during infusion of adenosine, lidocaine and magnesium (ALM), demonstrated by a decrease in urine output and creatinine clearance and an increase in plasma creatinine levels; however, this reversed after infusion was turned off. **(A)** Urine output during the study, measured hourly. **(B)** Plasma creatinine levels. **(C)** Creatinine clearance. **(D)** Urinary neutrophil gelatinase-associated lipocalin (NGAL)/creatinine ratio. *Significant difference at the end of the study. #Significantly different change over time between groups. †Significantly different development over time during infusion of ALM. ¥Significant difference before/after cessation ALM infusion. Data presented as median (95% confidence interval), except for creatinine clearance that is presented as mean (95% confidence interval). LPS, lipopolysaccharide.

Urine output decreased significantly during infusion of ALM (Figure 7A) but the production increased rapidly after ALM was discontinued, resulting in a significantly higher urine output in the ALM group when compared with controls at the end of the study. Despite these temporal differences, there was no significant difference in total urine production during the entire study (ALM, 487 (95% CI: 236 to 738) ml vs. control, 544 (95% CI: 300 to 788) ml). Plasma creatinine levels increased steadily in the ALM group during infusion (Figure 7B). After the infusion of ALM was discontinued, there was an immediate decrease in plasma creatinine. Creatinine levels remained 33% higher at the end of the study in the ALM group.

The higher plasma creatinine level during ALM infusion was due in part to decreased creatinine clearance. However, creatinine clearance was significantly higher in the ALM group when compared with controls after infusion was discontinued (Figure 7C). Both the urinary protein/creatinine ratio and NAGase/creatinine ratio increased in the ALM group during ALM infusion but returned to values comparable with the control group after infusion was turned off (Table 2). There was a significantly different development over time between groups with regards to the urinary NGAL/creatinine ratio; however, no significant group difference existed at the end of the study (Figure 7D). Overall markers of renal dysfunction increased in the ALM group during infusion of ALM, but returned to control group levels after the infusion, with the exception of higher plasma creatinine levels and an increase in creatinine clearance in the ALM group compared with controls.

## Discussion

In the present study, we showed in an endotoxemic porcine model that treatment with ALM improved cardiac function, increased the PaO_2_/FiO_2_ ratio with lower lung wet/dry ratios, and reduced inflammation indicated by lower TNFα levels and superoxide anion production. ALM therapy induced a transient hypotensive state and higher heart rates during infusion, with significantly higher oxygen delivery and lower whole body VO_2_ than controls. The hemodynamic status normalized immediately after discontinuation of therapy. In addition, ALM led to a transient drop in renal function during infusion that was reversed after the treatment was stopped.

### Adenosine, lidocaine and magnesium treatment

The treatment regime and dosing of ALM was determined from our published rat and porcine hemorrhage studies [10,13,20], and from pilot studies in the lipopolysaccharide porcine model. An intravenous bolus of ALM was administered at the start of lipopolysaccharide infusion as a loading dose to increase concentrations in the vascular compartment, followed by constant infusion. After 60 minutes, the ALM infusion dose was reduced to minimize further hypotension based on our pilot studies. Magnesium sulfate was added to AL (making ALM) based on its ability to improve hemodynamics and correct coagulopathy in a rat model of hemorrhagic shock [12,25].

This current study tested the combination ALM and not its individual components because in previous studies we have shown that it is the unique combination of ALM that exerts synergistic effects related to hemodynamic stability [12,25], myocardial salvage [20] and neutrophil activation [9], which were not conferred by the individual drugs alone.

### Hemodynamic response to ALM treatment

The current study was a proof-of-concept study with intriguing findings as organ function was improved during a transient pharmacologically induced hypotensive state. In the present study, ALM induced a reversible hypotensive state with MAP of 47 mmHg. We further showed that this hypotensive state was stable and associated with improvements in cardiac and pulmonary function, increased oxygen delivery, and normal lactate levels. Interestingly, using the same anesthesia and same size pigs, we have previously shown that a single bolus of ALM during resuscitation following hemorrhagic shock, despite the vasodilatory properties of each of its components [26-28], increased MAP from a shock state of 37 mmHg to ~48 mmHg with significantly lower blood lactate levels than controls [14]. Similarly, in the present study, despite MAP of 47 mmHg in normovolemic ALM pigs, cardiac function was improved and lactate levels were significantly lower than in controls at the end of the study. We conclude that the ALM-induced hypotensive state during lipopolysaccharide infusion had no signs of severe whole body ischemia with a balanced oxygen supply/demand.

Although the Surviving Sepsis Campaign guidelines endorse maintaining MAP greater than 65 mmHg [29], organ protection was observed despite the temporary pharmacologically induced hypotension during ALM infusion. It is important to distinguish between pathological hypotension and pharmacologically induced hypotension. Pathologically induced hypotension as a consequence of sepsis and systemic inflammation results in reduced tissue perfusion, increased lactate levels caused by adrenergic activation of glycolysis, a decrease in utilization and hypoxia, which if not corrected leads to organ dysfunction and increases mortality [30-32]*.* In contrast, pharmacologically induced hypotension as observed in the current study was associated with improved oxygen delivery and organ function. The concerns over inadequate tissue perfusion and energy supply/demand mismatch during hypotension in the septic patient may not be the same when hypotension is induced pharmacologically by drugs that maintain adequate organ and whole body oxygen supply/demand status and avoid anaerobic metabolism, and exert anti-inflammatory effects. Future studies are therefore warranted in which the hypotensive effects of the treatment with ALM are tested in models more closely representing the septic patient with hemodynamic instability.

Furthermore, whether the decrease in MAP is a potential protective mechanism or a potential side effect of the treatment needs to be further elucidated. It would be interesting to examine different doses of ALM on the effect of MAP with and without the infusion of vasopressors.

The heart rate was significantly higher in the ALM group compared with the control group at the end of the study, which may be detrimental because studies have demonstrated that heart rate >95 min^–1^ was associated with a higher mortality, leading to the use of beta blockers to improve outcome [33-35]. Higher heart rates in the present study are interesting because adenosine, lidocaine and Mg^2+^ individually possess negative chronotropic effects, as we have recently reported in the porcine model of hemorrhagic shock [14,36]. In our study, it appears that in the ALM group the positive chronotropic response caused by hypotension to maintain cardiac output and oxygen delivery overruled the known negative chronotropic effects of the individual drugs.

### Cardiac function

In the current study, lipopolysaccharide infusion impaired both systolic and diastolic function, and arterial–ventricular coupling. Systolic dysfunction was evident in controls by a rightward shift of the Ees and a decrease in d*P*/d*t*_max_ and the slope of the PRSW. Diastolic dysfunction was evident by an increase in Tau and d*P*/d*t*_min_. The present study did not investigate the cellular mechanisms of lipopolysaccharide-induced dysfunction, but these may include lipid peroxidation, abnormal calcium handling, production of inflammatory cytokines, and autonomic dysfunction [37]. Treatment with ALM resulted in a significant and clinically relevant improvement in all measured cardiac functional parameters after 5 hours of observation. The reduction in neutrophil activation and TNFα release with ALM may be a mechanism underlying cardioprotection as these mediators are known to depress myocardial function [38,39].

In our study, lipopolysaccharide infusion increased the Ea/Ees ratio in the control group over time as reported in other studies [40], which indicates a decrease in coupling efficiency and cardiac performance. This increase in the Ea/Ees ratio was prevented in the ALM group during the infusion period only. The decrease in SV and apparent loss in arterial–ventricular coupling efficiency observed in controls may be linked to a higher MPAP, and possibly right heart dysfunction contributing to a lower SV. Since Ees was unchanged in the ALM group, the lower Ea/Ees ratio in the ALM group was due largely to a significantly lower Ea (end systolic pressure/SV) relative to controls [41]. Hence, ALM optimizes arterial–ventricular coupling by reducing MAP and unloading the heart and by lowering MPAP and increasing SV.

### Pulmonary function

Intravenous administration of lipopolysaccharide is a widely used and relevant model of acute lung injury [23,42]. In the present study, acute lung injury was evident in controls by a decrease in the PaO_2_/FiO_2_ ratio, an increase in the alveolar–arterial oxygen difference, a higher MPAP, and an increase in the wet/dry ratio. Treatment with ALM improved the pulmonary status, manifested by a significantly higher PaO_2_/FiO_2_ ratio, a lower alveolar–arterial oxygen difference, lower MPAP, and a lower wet/dry ratio. At the end of the study, the PaO_2_/FiO_2_ ratio was 260 (95% CI: 221 to 299) in the control group and 388 (95% CI: 349 to 427) in the ALM group with a difference of 129 (95% CI: 73 to 184), which we regard as a clinically relevant difference. Following lipopolysaccharide infusion, pulmonary dysfunction and the increase in wet/dry ratio are most probably related to a combination of elevated microvascular pressure and increased vascular permeability [43].

The improvement in the wet/dry ratio and oxygenation with ALM treatment may relate to both a reduction in PVR and a reduction in vascular permeability. Kutzsche and colleagues showed in an endotoxemic porcine model that infusion of adenosine reduced extravascular lung water content without a reduction in MPAP [44], suggesting that the lower wet/dry ratio may in part be related to preserved endothelial integrity. Furthermore, Feng and colleagues have demonstrated that lidocaine alone attenuates acute lung injury through inhibition of nuclear factor-κΒ activation [45]. In our study, this is consistent with the observed significant decrease in TNFα production and leukocyte superoxide anion production, which are known mediators of endothelial dysfunction. However, treatment with ALM also caused a significant reduction in PVR, supporting our contention that the improvement in pulmonary function is related to both improved vascular permeability and a reduction in PVR.

### Acute kidney injury

Renal dysfunction is a common finding in septic patients, and previous animal studies have demonstrated that targeting a lower MAP resulted in a higher incidence of acute kidney injury [46], which is why we meticulously evaluated renal function using several parameters as additional impairment mediated by pharmacological induced hypotension may be of concern. Adenosine, for example, is believed to be involved in regulation of tubuloglomerular feedback, and infusion in humans increases renal blood flow and lowers the glomerular filtration rate [47,48]. The adenosine-mediated decrease in the glomerular filtration rate is mediated by A1 receptor activation and pre-glomerular vasoconstriction, whereas A2 receptor activation medicates post-glomerular arteriolar vasodilation reducing filtration pressure and cortical blood flow but preserving renal juxtamedullary blood flow [47-49]. In the present study, urine output and creatinine clearance decreased while plasma creatinine increased as a consequence of a reduced filtration pressure. During the ALM infusion, markers of tubular injury (NGAL and NAGase) may have increased as consequence of the lower MAP causing tubular ischemia. However, renal excretion of NGAL and NAGase normalized after the ALM treatment was discontinued, suggesting that minimal tubular injury occurred. Lower urine output may also be caused by a downregulation of tubular activity from the effect of adenosine or the detrimental effects of A3 receptor activation [50,51].

The 5-hour infusion period is too short to fully elucidate the effects of ALM infusion on tubular function, and future studies over longer times are required for a full renal assessment including histological evaluation.

### Oxygen consumption and delivery

Previous studies in septic patients have demonstrated that whole body VO_2_ is increased compared with that in healthy controls [52]. VO_2_ increased in the control group in the present study. In contrast, infusion of ALM maintained VO_2_ at a significantly lower set point than controls, along with significantly higher oxygen delivery and a higher arterial–venous oxygen difference_._ The VO_2_-lowering effect of ALM disappeared immediately after cessation of the infusion, indicating that the effect was directly related to the treatment. This is consistent with a previous study of porcine hemorrhagic shock in which the combination of adenosine and lidocaine reduced whole body VO_2_ by 27% after return of shed blood during resuscitation [13]. While most clinical trials have failed to improve the oxygen supply/demand by increasing supply, our study suggests that an alternative approach may be to use ALM infusion to lower demand [53,54].

In our study, it is possible that ALM reduced VO_2_ in part by blunting the hypermetabolic effects of elevated catecholamine levels via anti-adrenergic receptor modulation [55-57]. The potential anti-adrenergic effects of ALM may arise from adenosine’s well-known anti-adrenergic effect via activation of the A1 receptor [36,58] and magnesium’s effect to inhibit calcium channels at peripheral sympathetic nerve endings [59]. Further studies are required to examine this question *in vivo*. While plasma lactate levels increased in controls, lactate levels were consistently lower in the ALM group, consistent with an improved oxygen supply–demand balance. We recognize that the small difference in lactate levels may be clinically irrelevant; however, a recent clinical study demonstrated that even mild hyperlactatemia, similar to that observed in controls, was associated with worse outcome in critically ill patients [60].

### Limitations

This experimental porcine study of 5-hour continuous lipopolysaccharide infusion has several limitations that may limit its clinical translation. Firstly, continuous lipopolysaccharide infusion was chosen because it induces a rapid, reproducible systemic inflammatory response [18] and is a relevant model of acute lung injury [23,42]. The administration of ALM was started concomitant with lipopolysaccharide infusion, which does not reflect the clinical time course of delayed therapy after diagnosis of sepsis. The time course of lipopolysaccharide-induced immune activation is more rapid than the more gradual and prolonged natural time course in septic patients.

Secondly, clinical translation may be problematic since live bacteria were not used and the natural time course of organ failure normally occurs after 5 hours in humans, although recently it was demonstrated that ALM conferred significant protection in a rat model of cecal ligation and puncture [16].

Lastly, the hemodynamically stable porcine model without vascular co-morbidities, such as carotid stenosis and ischemic heart disease, is a model in direct contrast to the hemodynamically unstable patient suffering from severe sepsis or septic shock. The presence of vascular co-morbidities and hemodynamic instability may make these organs more vulnerable to hypoperfusion secondary to hypotension and offset the protective properties of ALM.

For translation from the current experimental model to the septic patient, the effect of ALM needs to be examined in a more clinically relevant model with live bacteria; hemodynamic instability and prolonged observation times with survival outcomes are required.

## Conclusion

The present study demonstrates that treatment with ALM in an endotoxemic porcine model: reduces leukocyte superoxide anion production and TNFα release; induces a state of reversible hypotension with improved oxygen delivery, cardiac function and pulmonary function; reduces whole body VO_2_; and causes a modest transient drop in renal function that is reversed after the treatment is stopped.

## Key messages

- Treatment with ALM induces a fully reversible stable hypotensive state.
- This hypotensive state is associated with increased oxygen delivery and heart rate, a decrease in oxygen consumption and lower lactate levels.
- During hypotension there is decrease in renal function that is fully reversed after treatment is turned off.
- Treatment with ALM improves cardiac and pulmonary function.
- Treatment with ALM attenuates TNFα levels and leukocyte superoxide anion production.

## Abbreviations

AL: adenosine and lidocaine
ALM: adenosine, lidocaine and magnesium
CI: confidence interval
d*P*/d*t*_max_: maximum rate of pressure development over time
d*P*/d*t*_min_: maximum rate of pressure decrease over time
Ea: arterial elastance
Ees: end systolic elastance (end systolic pressure–volume relationship)
IL: interleukin
FiO_2_: inspired fraction of oxygen
MAP: mean arterial pressure
MPAP: mean pulmonary arterial pressure
NAGase: *N*-acetyl-β-d-glucosaminidase
NGAL: neutrophil gelatinase-associated lipocalin
PaO_2_: arterial partial pressure of oxygen
PRSW: preload recruitable stroke work
PVR: pulmonary vascular resistance
SV: stroke volume
Tau: time constant of isovolumic relaxation
TNFα: tumor necrosis factor alpha
VO_2_: oxygen consumption
